# Immune checkpoint inhibitor-related pancreatitis: a comprehensive review of epidemiology, pathophysiology, and management

**DOI:** 10.1007/s00535-026-02389-2

**Published:** 2026-04-01

**Authors:** Yoonchan Lee, Dongwook Oh, Sung Hyun Cho, Seung-Mo Hong, Tae Jun Song

**Affiliations:** 1 Department of Gastroenterology, Asan Medical Center, University of Ulsan College of Medicine, Seoul, Republic of Korea; 2 Department of Pathology, Asan Medical Center, University of Ulsan College of Medicine, Seoul, Republic of Korea

**Keywords:** Immune checkpoint inhibitor, Pancreatitis, Autoimmune pancreatitis, Immune-related adverse events

## Abstract

Immune checkpoint inhibitors (ICIs) have revolutionized cancer therapy but are associated with immune-related adverse events (irAEs) affecting multiple organ systems. ICI-related pancreatitis (ICI-P) is an uncommon but clinically significant toxicity with potential for irreversible pancreatic damage. This review synthesizes current evidence on the epidemiology, pathophysiology, diagnosis, and management of ICI-P. The incidence ranges from 0.5% to 5.7% depending on definitions and regimens, with combination immunotherapy conferring a significantly higher risk. Histopathologically, ICI-P is characterized by acinar-centric T cell infiltration with temporal evolution from CD4 + to CD8 + predominance. Most cases present as asymptomatic lipase elevation rather than clinical pancreatitis; however, long-term sequelae, including pancreatic atrophy (44–55%), new-onset diabetes mellitus, and exocrine insufficiency, are increasingly recognized. The role of corticosteroids remains controversial, with evidence suggesting efficacy for acute symptoms but uncertain impact on long-term outcomes. Notably, ICI-P development may be associated with improved overall survival, potentially reflecting enhanced antitumor immunity. This review provides an evidence-based framework for diagnosis and management while highlighting unresolved controversies requiring further investigation.

## Introduction

Immune checkpoint inhibitors (ICIs) have fundamentally transformed the landscape of cancer therapeutics over the past decade. These agents act by blocking inhibitory T-cell pathways, thereby enhancing antitumor immune responses [1, 2]. The primary targets of currently approved ICIs include cytotoxic T-lymphocyte–associated protein 4 (CTLA-4), programmed cell death protein 1 (PD-1), and programmed death-ligand 1 (PD-L1). To date, eleven ICI agents have received U.S. Food and Drug Administration approval: ipilimumab and tremelimumab (anti-CTLA-4), nivolumab, pembrolizumab, cemiplimab, dostarlimab, toripalimab, and retifanlimab (anti-PD-1), and atezolizumab, durvalumab, and avelumab (anti-PD-L1) [3, 4].

The clinical success of ICIs has been accompanied by a distinct spectrum of immune-related adverse events (irAEs), reflecting nonspecific immune activation beyond the tumor microenvironment [5]. Gastrointestinal irAEs are among the most frequently reported after cutaneous toxicities and encompass a wide range of severity [6]. Within this spectrum, pancreatic involvement has emerged as an increasingly recognized but relatively uncommon complication. Immune checkpoint inhibitor–related pancreatitis (ICI-P) represents a heterogeneous entity that differs in several respects from classical forms of autoimmune pancreatitis (AIP), with accumulating evidence suggesting distinct clinical, radiologic, and histopathologic characteristics [7–10].

This review provides a comprehensive and critical synthesis of current evidence on the epidemiology, pathophysiology, clinical manifestations, diagnosis, and management of ICI-P, with particular emphasis on recent advances, while highlighting areas of ongoing debate, including disease classification, therapeutic strategies, and prognostic implications.

## Nomenclature and clinical spectrum of immune-mediated pancreatitis

The pancreas is inherently susceptible to immune-mediated damage. Traditionally, autoimmune pancreatitis has been classified into two established subtypes: type 1 AIP, characterized by IgG4-positive plasma cell infiltration as part of IgG4-related disease, and type 2 AIP, defined by granulocytic epithelial lesions (GEL) and occasionally associated with inflammatory bowel disease [11, 12]. The advent of ICI therapy has introduced a distinct form of immune-mediated pancreatic injury, which some investigators have proposed designating as type 3 autoimmune pancreatitis [7–9].

However, the nomenclature of this entity remains debated. Proponents of the type 3 AIP designation emphasize its immune-mediated mechanism and distinctive clinicopathologic features, arguing for classification alongside established AIP subtypes [7–9]. In contrast, Chen et al. have suggested that the term ICI-P more appropriately reflects its drug-induced etiology while maintaining consistency with nomenclature used for other immune-related adverse events (e.g., ICI-related hepatitis, ICI-associated colitis) [10]. Accordingly, until the precise pathobiological relationship between ICI-P and classical AIP subtypes is better defined, this review adopts the term ICI-P while acknowledging the ongoing conceptual discussion.

Importantly, the classification of ICI-P as a true autoimmune pancreatitis subtype remains provisional. Unlike type 1 or type 2 autoimmune pancreatitis, ICI-P lacks hallmark features such as IgG4-related systemic involvement, GEL as a defining histologic criterion, and a reproducible pattern of sustained steroid responsiveness. These distinctions suggest that ICI-P may represent a distinct form of immune-mediated, drug-induced pancreatitis that overlaps with—but is not identical to—classical AIP entities (Table 1).

**Table 1:** Comparison of Immune-Mediated Pancreatitis Subtypes

Feature	Type 1 AIP	Type 2 AIP	ICI-P (Type 3 AIP)
Age/Gender	Older males (60–70 s)	Younger, equal gender	Cancer demographics (58–77 yrs); slight male predominance
Serology	Elevated IgG4 (80–90%)	Normal IgG4	Normal IgG4; rare IgG4 + cells (< 1/HPF)
Histology	IgG4 + plasma cells, LPSP, obliterative phlebitis	GEL, neutrophil infiltration, periductal	Acinar-centric, CD3 + T cells, fibrosis, ADM; no obliterative phlebitis
T cell profile	CD4 + predominant	Variable	Time-dependent: CD4 + early → CD8 + late
Extrapancreatic	IgG4-related disease	IBD association	Other irAEs (55–65%): hepatitis, colitis, thyroid
Steroid response	Excellent	Excellent	Partial/controversial

*ADM* acinar-to-ductal metaplasia, *AIP* autoimmune pancreatitis, *GEL* granulocytic epithelial lesion, *HPF* high-power field, *IBD* inflammatory bowel disease, *LPSP* lymphoplasmacytic sclerosing pancreatitis

Clinically, ICI-P encompasses a broad spectrum of pancreatic involvement, ranging from asymptomatic enzyme elevation to overt acute pancreatitis and autoimmune pancreatitis-like presentations with diffuse pancreatic enlargement. Given that subclinical pancreatic involvement may evolve into irreversible atrophy, this review adopts the term ICI-P broadly to include cases with lipase elevation ≥ 3 × the upper limit of normal (ULN) after exclusion of alternative etiologies [6, 7].

## Epidemiology and risk factors

The reported incidence of ICI-P varies considerably across studies, largely reflecting differences in definitions, surveillance intensity, and patient populations. In meta-analyses of randomized controlled trials, the overall incidence of ICI-related pancreatic events with monotherapy ranges from approximately 1% to 4% [10, 16–18]. When distinguishing between clinical pancreatitis and biochemical abnormalities, clinical pancreatitis occurs in approximately 0.9–1.9% of patients, whereas asymptomatic lipase elevation is observed in 1.3–4.2% [6, 17]. Single-center and multicenter cohort studies from the United States and Asia have yielded comparable results, with biochemical pancreatic injury ranging from 1.8% to 4.9% and clinical pancreatitis occurring in 0.5–1.1% [4, 13, 19, 20]. One study reported substantially higher rates of any-grade enzyme elevation (46.5%), likely reflecting intensive surveillance protocols, though clinical pancreatitis per National Comprehensive Cancer Network (NCCN) criteria was observed in only 5.7% [21].

The incidence of ICI-P varies significantly based on treatment regimen and cancer type. Anti-CTLA-4 monotherapy carries nearly triple the risk compared with anti-PD-1 therapy [10, 22]. Combined ICI therapies with both CTLA-4 and PD-1 inhibitors carry a much higher risk than monotherapy, ranging from 2 to 10% [18, 22]. Simultaneous combination therapy (nivolumab + ipilimumab) demonstrates significantly higher grade ≥ 3 ICI-P rates compared to both monoimmunotherapy (23.1% vs. 7.9%, p < 0.001) and sequential dual therapy (23.1% vs. 10.0%, p = 0.032), suggesting that timing of combination affects toxicity risk [21]. Among cancer types, patients with melanoma have higher incidences of pancreatic involvement (3.7% vs. 1.2%), with lipase elevation reaching 13.5% in one multicenter study [22, 23].

The largest real-world pharmacovigilance analysis to date, identifying 606 cases from the FDA Adverse Event Reporting System database (March 2011–September 2024), provides further insight into drug-specific signals [18]. Among ICI classes, PD-1 inhibitors accounted for the majority of cases (62%), followed by PD-L1 inhibitors (23%) and CTLA-4 inhibitors (15%), though this distribution largely reflects prescribing patterns. Among specific agents, pembrolizumab and nivolumab were most commonly implicated (31% and 30%, respectively), while cemiplimab demonstrated the strongest reporting signal (reporting odds ratio [OR] 151.20) [18].

Several additional patient- and treatment-related risk factors have been identified through multivariable analyses. Treatment-related risk factors include CTLA-4 inhibitor use (OR 21.27), combination ICI therapy (OR 27.94), ≥ 10 ICI cycles, and prior interferon therapy (OR 13.53), whereas prior cytotoxic chemotherapy appears protective (OR 0.18), possibly due to immunocompromised status reducing autoimmune-like reactions [13, 20, 21]. Among patient-related factors, certain cancer types confer increased risk, particularly renal cell carcinoma (OR 7.33) and melanoma (OR 4.96) [20]. Elevated baseline serum amylase (≥ 70 U/L) has emerged as a novel independent risk factor (OR 6.10), suggesting that patients with higher baseline exocrine function may be more susceptible to T cell-mediated pancreatic injury [13]. Concurrent irAEs in other organs also significantly increase risk (OR 9.00), particularly hepatic (OR 6.36) and endocrine disorders (OR 6.62) [20].

Pre-existing autoimmune disease represents another independent risk factor for irAE development, associated with both increased irAE occurrence (adjusted OR 2.52) and earlier onset (median irAE-free survival: 5.7 vs. 10.4 months) [39]. Notably, de novo irAEs were more frequent than flare-ups of pre-existing conditions, suggesting that autoimmune disease confers a non-specific risk for immune-related toxicity rather than simply predisposing to disease flares.

## Pathophysiology

The pathophysiology of ICI-P involves a distinct mechanism of immune-mediated pancreatic injury that differs fundamentally from classical autoimmune pancreatitis subtypes. While type 1 AIP is characterized by IgG4-positive plasma cell infiltration and type 2 AIP by neutrophilic epithelial lesions, ICI-P demonstrates predominantly T cell-mediated acinar cell injury [7–9, 24].

The proposed mechanism begins with the disruption of immune tolerance following checkpoint blockade. By releasing inhibitory signals on T cells, ICIs may unmask autoreactive T cell clones that target pancreatic antigens [7–9]. Supporting the immune activation hypothesis, checkpoint-blocker-induced autoimmunity has been associated with favorable antitumor outcomes and distinct T-cell expression profiles, suggesting that irAEs and therapeutic efficacy share overlapping immunologic mechanisms [26]. Additional factors influencing susceptibility include the intestinal microbiota, particularly the Bacteroidetes to Firmicutes ratio, and human leukocyte antigen variation, suggesting both environmental and genetic components to ICI-P risk [6, 25].

The histopathologic consequences of this immune dysregulation have been characterized in the most detailed study available to date. This study, examining a limited number of cases (5 specimens from 4 patients), identified acinar-centric mixed inflammatory infiltration as the predominant finding (100%), distinguishing ICI-P from type 2 AIP, which characteristically shows periductal inflammation [10]. Progressive tissue remodeling was evident, including loss of acinar tissue (80%), pancreatic fibrosis with prominent fibroblasts and myofibroblasts (80%), and acinar-to-ductal metaplasia (80%). Prominent edema (80%) appears to be a consistent early feature. GEL-like duct injury with neutrophilic infiltration was observed in 40% of specimens, representing a potential overlap with type 2 AIP; however, pancreatic ducts are typically not visualized in core needle biopsies, limiting the diagnostic utility of this finding [10]. Importantly, hallmarks of type 1 AIP were absent or rare: no obliterative phlebitis or granulomas were identified, storiform fibrosis was seen in only one patient, and IgG4-positive plasma cells were sparse (< 1 cell per HPF), clearly distinguishing ICI-P from IgG4-related disease [10]. The predominant histologic findings are summarized in Table 2.

**Table 2:** Histopathologic Features of ICI-Related Pancreatitis

Histologic Finding	Frequency	AIP Type
Acinar-centric mixed inflammatory infiltrate	100%	Distinct
Atrophy (loss of acinar cells)	80%	–
Fibrosis	80%	–
Acinar-to-ductal metaplasia	80%	–
Edema	80%	–
GEL-like duct injury	40%	Type 2-like
Storiform fibrosis	20%	Type 1-like
Obliterative phlebitis	0%	Type 1
Granulomas	0%	–
IgG4 + plasma cells (> 10/HPF)	0%	Type 1

*GEL* granulocytic epithelial lesion, *HPF* high-power field

**Fig. 1:**
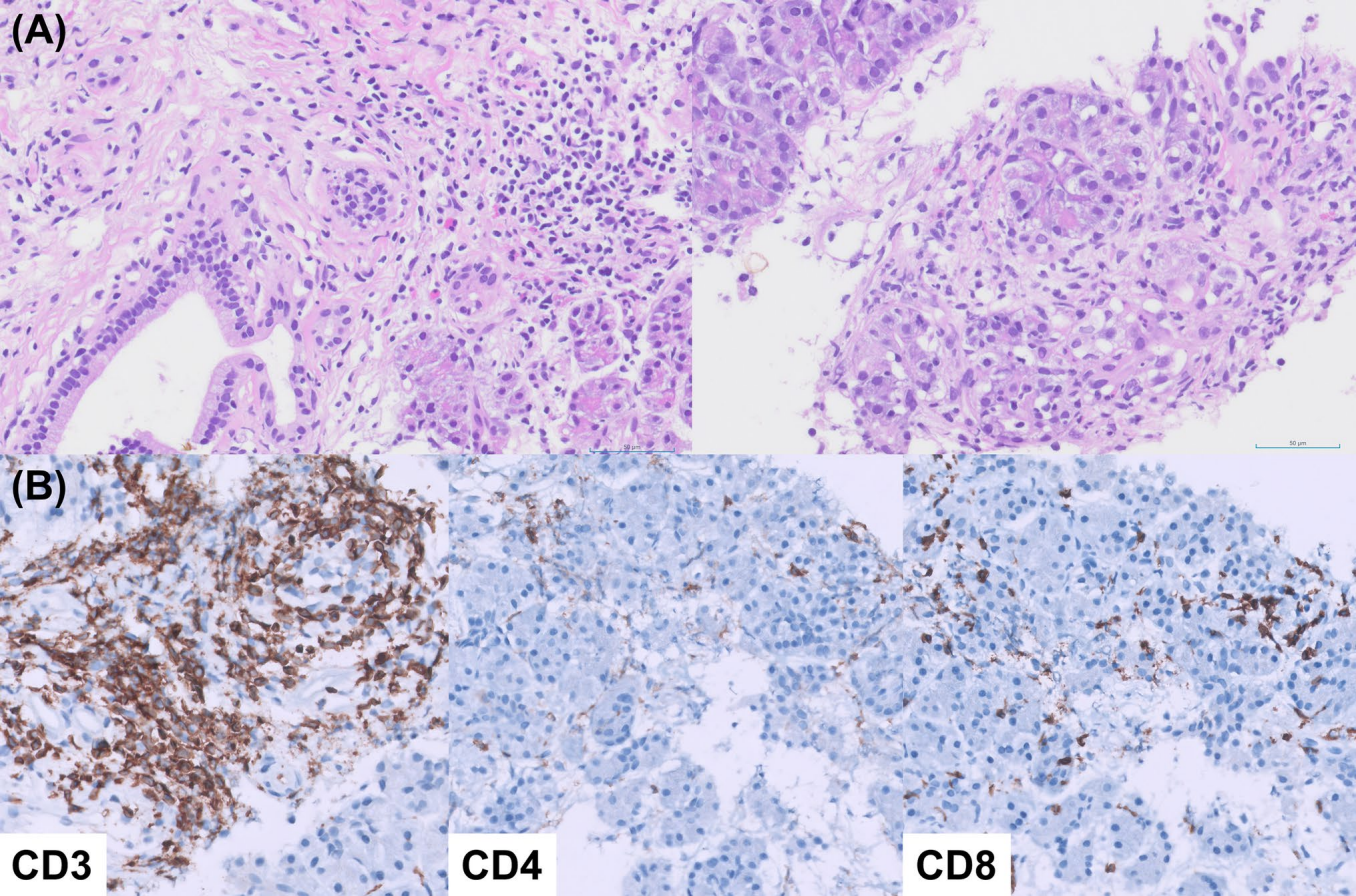
Histopathologic Features of ICI-Related Pancreatitis. Representative histopathologic findings from reported cases of ICI-related pancreatitis. **A** Acinar-to-ductal metaplasia with lymphocytic infiltrate, demonstrating acinar injury and tissue remodeling characteristic of ICI-P. **B** CD3, CD4, and CD8 immunohistochemistry showing T cell–predominant inflammation with temporal evolution from CD4 + to CD8 + predominance

Immunohistochemical characterization has established that the inflammatory infiltrate is predominantly composed of CD3 + T cells, with CD8 + granzyme B + cytotoxic T lymphocytes playing a central role [10, 13, 21]. Multiplex fluorescence immunohistochemistry has demonstrated abundant granzyme B-positive CD8 + T cell infiltration in the pancreatic parenchyma, providing direct evidence for cytotoxic injury to acinar cells [13]. Endoscopic ultrasound (EUS)-guided fine needle biopsy of peripancreatic lymph nodes has confirmed greater infiltration of CD8 + than CD4 + lymphocytes, consistent with irAE pathology [21]. Representative histopathologic and immunohistochemical findings are shown in Fig. 1.

Intriguingly, the CD4 +/CD8 + ratio appears to evolve in a time-dependent manner, suggesting distinct phases of disease progression [10]. In early specimens (6–10 days post presentation), CD4 + T cells predominated with CD4 +/CD8 + ratios of 2.4–2.6, suggesting an initial helper T cell-driven phase. By day 49, approximately equal numbers of CD4 + and CD8 + T cells were observed (ratio 1.1). In an autopsy specimen obtained 54 days post presentation, CD8 + T cells markedly predominated (ratio 0.1). This temporal evolution suggests that CD4 + T cells may orchestrate the early immune response, with subsequent transition to CD8 + cytotoxic T cell predominance driving progressive acinar destruction and eventual parenchymal atrophy. This model aligns with the expected mechanism of PD-1/PD-L1 inhibition and may explain the chronic sequelae observed in a subset of patients.

## Clinical manifestations

The majority of ICI-P cases manifest as asymptomatic pancreatic enzyme elevation rather than classical acute pancreatitis. Across cohorts, only approximately 30–40% of patients develop overt pancreatitis with abdominal pain, nausea, and vomiting, whereas 30–63% present with isolated lipase elevation without symptoms [6, 10]. In the Fukui cohort, 87.5% of patients had pancreatic injury without clinical pancreatitis, and only 12.5% developed symptomatic disease [13].

When symptomatic, ICI-P presents similarly to classic pancreatitis, with abdominal and/or back pain, nausea and vomiting, fever, and diarrhea [6]. Patients typically present with severe epigastric pain that may radiate to the back, and physical examination may reveal abdominal tenderness, distention, hypoactive bowel sounds, or jaundice. In severe cases, systemic manifestations including tachypnea, hypoxemia, tachycardia, or hypotension may occur [27]. Although ICI-P generally follows a mild and favorable clinical course, severe life-threatening cases have been reported, including fatal acute pancreatitis [28].

The median time to onset of ICI-P varies widely across studies, with reported medians ranging from 92 to 108 days (overall range 3–1576 days) [10, 13, 18, 20]. The median number of ICI administrations before onset was 3 (range 1–50) [13]. Notably, ICI-P can develop after treatment discontinuation; in one multicenter study, 5 of 19 patients developed ICI-P following ICI cessation [20]. This wide variability in onset timing underscores the need for continued vigilance throughout and beyond the course of ICI therapy.

A notable feature of ICI-P is the high frequency of concurrent immune-related adverse events, observed in 55–79% of patients across studies [13, 18, 20]. A dose–response relationship exists between ICI-P severity and other organ involvement: 85.7% of patients with grade ≥ 3 ICI-P experienced grade ≥ 3 irAEs in other organs, compared to 26.8% in grade 2 ICI-P patients and 10.8% in patients without ICI-P (p < 0.001) [13]. The most common concurrent irAEs include endocrine disorders (42.1%), particularly hypothyroidism/thyroiditis and adrenal insufficiency, as well as hepatotoxicity (31.6%) [13, 20]. In severe cases, multiple concurrent irAEs may occur, including myositis, myocarditis, hepatitis, and colitis [10]. This observation emphasizes the importance of comprehensive multi-organ evaluation in patients diagnosed with ICI-P.

## Diagnosis and severity assessment

Current guidelines do not recommend routine monitoring of amylase and lipase levels in asymptomatic patients receiving ICIs [6, 29]. Nevertheless, lipase measurement may be warranted in patients presenting with other immune-related toxicities, given that 55–65% of ICI-P cases occur concurrently with other irAEs [13, 18]. Notably, only 29% of hyperlipasemia cases in patients receiving ICIs are attributable to ICI use, with the majority resulting from alternative causes such as malignant obstruction, bowel ischemia, or extrapancreatic irAEs [15]. This underscores that ICI-P remains fundamentally a diagnosis of exclusion [7]. Additionally, elevated baseline serum amylase prior to ICI initiation has been identified as an independent risk factor for subsequent ICI-P, suggesting that pretreatment enzyme levels may help stratify susceptibility [13]. During clinical presentation, laboratory abnormalities typically include elevations in serum amylase and lipase, with lipase being the more sensitive marker.

**Fig. 2:**
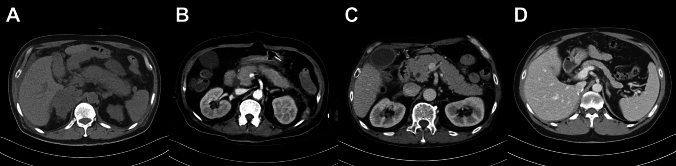
Representative imaging findings of ICI-related pancreatitis. **A** Contrast-enhanced CT showing diffuse pancreatic enlargement with peripancreatic fat stranding, consistent with acute interstitial pancreatitis pattern (80% of cases). **B** Focal pancreatic tail enlargement with subtle peripancreatic inflammation. **C** Mass-like enlargement of pancreatic head mimicking malignancy (AIP-like pattern). **D** Post-pancreatitis pancreatic atrophy on follow-up imaging

Imaging abnormalities are not universally present in ICI-P; in one cohort of patients with significant lipase elevation (≥ 5 × ULN), only 20% demonstrated imaging findings consistent with pancreatitis, while the remainder had normal pancreatic parenchyma [21]. When abnormalities are present, the most common CT or MRI findings include diffuse or focal pancreatic enlargement resembling acute interstitial pancreatitis or autoimmune pancreatitis, with the largest systematic imaging study (n = 25) identifying two distinct radiological patterns: acute interstitial pancreatitis (80%) and autoimmune pancreatitis-like (16%) [10, 24] (Fig. 2). Necrotizing pancreatitis and pseudocyst formation are rare, and most cases have CT severity index scores < 3, consistent with mild acute pancreatitis [21, 24]. Notably, approximately 16% of patients exhibit focal mass-like pancreatic enlargement, and on 1⁸F-FDG PET/CT, focal uptake (66%) can mimic metastatic disease, underscoring the importance of clinical correlation when evaluating pancreatic lesions during oncologic surveillance [10, 24]. Biopsy is generally not required but may be considered for atypical presentations or to exclude malignancy; when performed, EUS-guided fine-needle biopsy can confirm the characteristic T cell-predominant infiltrate and rule out neoplasia [6, 10, 13, 30]. However, ICI-P lacks pathognomonic histologic features and shows considerable overlap with other forms of pancreatitis, particularly on small biopsy specimens, necessitating careful correlation with the patient's medication history when nonspecific chronic pancreatitis patterns are encountered [10]^.^

The diagnosis of ICI-P should be based on the following criteria: documented exposure to an immune checkpoint inhibitor, lipase elevation ≥ 3 × the ULN, and exclusion of other causes including gallstones, alcohol, hypertriglyceridemia, other drug-induced pancreatitis, viral infection, and malignancy [7, 18]. Improvement after ICI withdrawal and/or corticosteroid therapy supports the diagnosis but is not required for initial clinical decision-making. Severity classification varies according to the grading system applied: the Common Terminology Criteria for Adverse Events (CTCAE) version 5.0 grades pancreatic adverse events based on enzyme elevation levels and clinical symptoms (Table 3), whereas the NCCN classification integrates enzyme elevation, radiologic findings, and clinical symptoms to stratify severity into mild, moderate, and severe categories (Table 4) [40]. Several investigators have noted that these frameworks may inadequately capture organ failure and complications, advocating instead for the modified Marshall scoring system [7–9]. Given that many studies apply the Revised Atlanta criteria, a standardized severity grading system specific to ICI-P remains an unmet need [14, 19].

**Table 3:** Common Terminology Criteria for Adverse Events (CTCAE) Version 5.0: Pancreatic Adverse Events

Adverse Event	Grade 1	Grade 2	Grade 3	Grade 4	Grade 5
Pancreatitis	–	Enzyme elevation; radiologic findings only	Severe pain, vomiting; medical intervention indicated	Life threatening consequences; urgent intervention indicated	Death
Lipase	> 1.0–1.5 × ULN	> 1.5–2.0 × ULN; > 2.0–5.0 × ULN and asymptomatic	> 2.0–5.0 × ULN with symptoms; > 5.0 × ULN and asymptomatic	> 5.0 × ULN with symptoms	–
Amylase	> 1.0–1.5 × ULN	> 1.5–2.0 × ULN; > 2.0–5.0 × ULN and asymptomatic	> 2.0–5.0 × ULN with symptoms; > 5.0 × ULN and asymptomatic	> 5.0 × ULN with symptoms	–

*ULN* upper limit of normal

**Table 4:** National Comprehensive Cancer Network (NCCN) Grading of Immune Checkpoint Inhibitor-Associated Pancreatitis

Grade	Description
Mild (Grade 1)	Elevation of amylase/lipase > 3 × ULN or radiologic findings on CT or clinical findings consistent with pancreatitis
Moderate (Grade 2)	Two of three: elevation of amylase/lipase > 3 × ULN + radiologic findings on CT + clinical findings concerning for pancreatitis
Severe (Grades 3–4)	Elevation of amylase/lipase + radiologic findings + severe abdominal pain or vomiting and hemodynamically unstable

*CT* computed tomography, *ULN* upper limit of normal

Overall, the diagnosis of ICI-P is often challenging due to atypical imaging and histologic findings, variable clinical courses, and the need to establish a causal relationship with ICI therapy. Consequently, the diagnosis ultimately relies on clinical judgment integrating imaging, pathology, and clinical course, highlighting the need for disease-specific diagnostic algorithms and severity criteria.

## Management

**Fig. 3:**
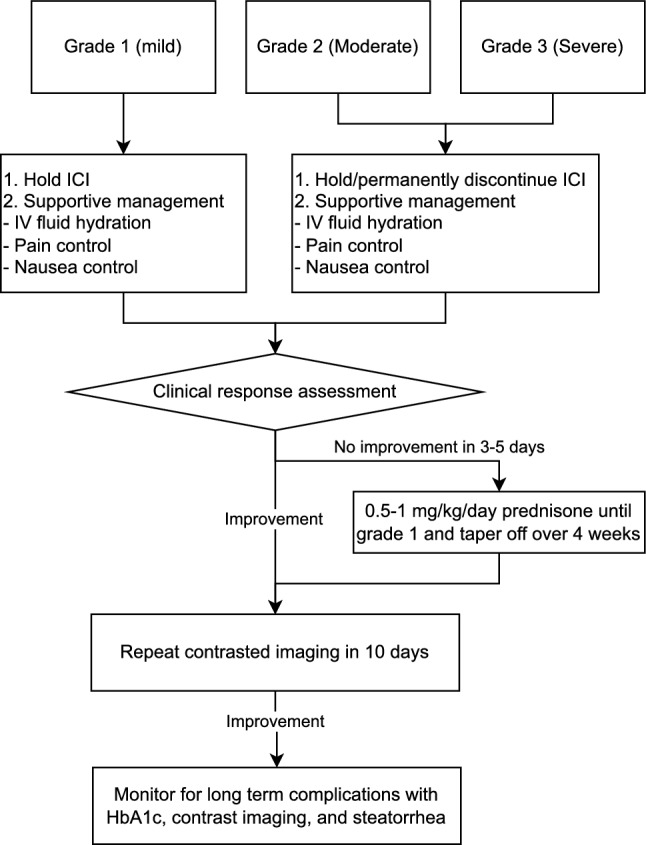
Management Algorithm for ICI-Related Pancreatitis. Management is guided by severity grading adapted from the National Comprehensive Cancer Network (NCCN) classification. Initial evaluation includes exclusion of other causes and contrast-enhanced abdominal imaging. For all grades, supportive care consists of intravenous fluid hydration, pain control, and antiemetic therapy. In mild (grade 1) cases, ICI therapy may be held with close monitoring. In moderate (grade 2) to severe (grade 3) cases, ICI should be held or permanently discontinued. Clinical response is assessed within 3–5 days; if no improvement is observed, corticosteroids (prednisone 0.5–1 mg/kg/day) are initiated and continued until symptoms improve to grade 1, followed by tapering over 4 weeks. After clinical improvement, contrast-enhanced imaging is repeated at 10 days. Long-term monitoring includes periodic HbA1c measurement, contrast-enhanced imaging, and evaluation for steatorrhea to detect endocrine and exocrine insufficiency. ICI, immune checkpoint inhibitor; IV, intravenous; HbA1c, hemoglobin A1c

Management of ICI-P follows a graded approach based on severity (Fig. 3) [7, 40]. For mild (grade 1) cases, ICI therapy may be held with supportive management. For moderate (grade 2) to severe (grade 3) cases, ICI should be held or permanently discontinued [29, 31]. When ICI-P presents with clinical features of acute pancreatitis, management should follow established acute pancreatitis guidelines. Supportive care remains the cornerstone of treatment, including intravenous fluid administration with goal-directed fluid resuscitation (5–10 mL/kg/h or less than 4.1 L over initial 24 h) using balanced solutions such as Lactated Ringer's, along with pain control and antiemetic therapy [32]. Early enteral nutrition is associated with decreased incidence of infection [32]. Clinical response should be assessed within 3–5 days; if no improvement is observed, corticosteroids (prednisone 0.5–1 mg/kg/day) may be initiated and continued until symptoms improve to grade 1, followed by tapering over 4 weeks.

The role of corticosteroids in ICI-P management remains controversial. A systematic review found that 73.8% of patients received corticosteroid therapy, with an overall improvement rate of 83.6% [18]. However, the largest single-center study from MD Anderson (229 patients) found that corticosteroids had no impact on shortening the acute phase, preventing long-term adverse outcomes, or improving overall survival [6, 33]. Furthermore, steroid use in immunocompromised patients with advanced malignancies poses a significantly increased risk of infectious events, particularly when administered for longer than 30 days [7]. Collectively, available evidence suggests that corticosteroids may be effective for controlling acute inflammatory manifestations but do not substantially alter the immediate clinical course or long-term outcomes. Therefore, while corticosteroids remain appropriate for symptomatic disease, their routine use in asymptomatic or mild cases is not supported by current evidence.

For steroid-refractory ICI-P, options including infliximab, rituximab, mycophenolate mofetil, and azathioprine have been reported, although evidence remains limited to case reports and small series [34–37]. The risks of worsening irAEs must be balanced with the immunosuppressants' impact on ICI anti-cancer efficacy and the increased risk of infection in patients with advanced malignancies.

## Long-term outcomes and prognosis

ICI-P represents a rare but potentially irreversible autoimmune injury to the exocrine pancreas that can lead to permanent pancreatic atrophy and dysfunction [7]. A systematic review reported overall outcomes of 83.6% improvement, 8.2% recurrence, and 8.2% death [18]. However, improvement in acute symptoms does not preclude long-term sequelae. Up to 15% of patients develop chronic features including recurrent acute pancreatitis, chronic pancreatitis with calcifications, and exocrine insufficiency [6, 10]. Post-pancreatitis pancreatic atrophy has been reported in 44% of patients, with 36% of those developing exocrine or endocrine insufficiency [24]. Over 55% of patients demonstrate > 20% pancreatic volume loss at 1-year follow-up, and new-onset insulin-dependent diabetes mellitus has been reported in 0.2–19% of patients, reflecting autoimmune destruction of islet cells [6, 10]. Rarely, isolated exocrine pancreatic insufficiency may occur [6]. Follow-up imaging typically shows significant decrease in pancreatic thickness with persistent loss of normal pancreatic T1 signal intensity and progressive contrast enhancement, likely reflecting post-inflammatory fibrosis [10]. While these long-term sequelae are irreversible, they can be managed with oral pancreatic enzyme supplements and/or hypoglycemic agents [7]. Accordingly, close monitoring for symptoms of pancreatic insufficiency, including hyperglycemia and steatorrhea, is indicated for all patients with ICI-P history.

An intriguing finding from recent studies is the potential association between ICI-P development and improved oncologic outcomes. The Fukui cohort demonstrated that patients with ICI-P had significantly better overall survival than those without ICI-P (median: not reached vs. 490 days; p < 0.001), validated by landmark analyses at 3, 6, and 12 months [13]. The conditional survival rates were 88.9% versus 57.9% at 1 year, and 77.5% versus 41.2% at 2 years, for patients with versus without ICI-P [13]. Furthermore, patients who developed irAEs in multiple organs (≥ 2) had significantly better overall survival than those with single-organ irAEs or no irAEs, a finding consistent with the broader literature suggesting that irAE development may reflect enhanced antitumor immunity [13, 38]. Subgroup analyses demonstrated that this survival benefit was observed across multiple cancer types and ICI regimens, including PD-1 monotherapy, PD-L1 monotherapy, and combination therapy [13]. The mechanism underlying this association remains unclear, and ICI-P should be interpreted as a potential biomarker of immune activation rather than a direct contributor to improved survival.

## ICI rechallenge

Data on ICI rechallenge following ICI-P are limited but generally reassuring. Across multiple studies, recurrence rates following rechallenge appear low. In the Japanese multicenter study, among 14 patients with ICI-P improvement, 6 were rechallenged, and only 1 (16.7%) experienced relapse, which improved with ICI discontinuation and steroid therapy [20]. A systematic review found that 13.7% were able to restart ICI therapy, and among 7 patients who were rechallenged, none experienced recurrence of pancreatitis [18]. Similarly, 5 of 10 patients in the Nagoya cohort underwent ICI rechallenge without pancreatitis recurrence [4], although one case of relapse after rechallenge was reported in the Fukui cohort [13]. In this cohort, all six patients with ICI-related pancreatitis discontinued ICI therapy, and five received steroid therapy, resulting in clinical improvement in all cases. Among patients with ICI-P without clinical pancreatitis, 17 discontinued therapy while 25 continued ICI treatment under careful monitoring, with all patients achieving improvement [13]. These data suggest that ICI rechallenge may be safely considered in selected patients with resolved ICI-P, though careful monitoring is essential.

## Conclusions

ICI-P has emerged as a distinct clinical entity characterized by T cell-mediated pancreatic injury with unique histopathologic features, including acinar-centric inflammation and temporal evolution of CD4 +/CD8 + ratios. Unlike classical autoimmune pancreatitis, ICI-P frequently presents as asymptomatic enzyme elevation, yet carries significant risk of long-term sequelae, including pancreatic atrophy, diabetes mellitus, and exocrine insufficiency. The association between ICI-P and improved overall survival suggests that pancreatic injury may reflect heightened systemic immune activation, though causality remains unestablished.

Several controversies warrant further investigation. First, the nomenclature remains unsettled; while "type 3 AIP" emphasizes immune-mediated pathogenesis, "ICI-related pancreatitis" more accurately reflects its drug-induced etiology. Second, the role of corticosteroids beyond acute symptom control is unclear, with current evidence suggesting limited impact on disease course or long-term outcomes. Third, the clinical significance of asymptomatic lipase elevation and optimal monitoring strategies remain undefined. Finally, recently identified risk factors—elevated baseline amylase and concurrent irAEs—require validation in independent cohorts.

As ICI indications expand and combination regimens become standard, the incidence of ICI-P is expected to rise. A multidisciplinary approach involving oncology, gastroenterology, pathology, and endocrinology is essential for optimal management. Addressing current knowledge gaps will require prospective studies integrating clinical, immunologic, radiologic, and histopathologic data to establish disease-specific diagnostic criteria, severity grading systems, and evidence-based treatment protocols.
